# Cyst in the Uterus of a Child Three Months Old, with Another Cyst like a Corpus Luteum in One of the Ovaries

**Published:** 1848-01

**Authors:** 


*Cyst in the Uterus of a child three months old, with another cyst like a corpus luteum in one of the ovaries.*—Dr. T. M. Lee showed to the Society the uterus and ovaries of a child that had died of an acute disease after a few hours' illness, and had previously exhibited no symptoms which indicated any derangement of the genito-urinary organs. It was during an examination of the body after death, that a vascular protuberance was discovered on one of the ovaries, similar to that which is observed when a corpus luteum is present. A section of this ovary disclosed a cyst corresponding to the bulge upon its surface, having a dense fibrous capsule, thinly coated with a yellow curdy matter, and containing serous fluid. Both the ovary and Fallopian tube of this side were much larger than those of the other side. When the vagina and the uterus were slit open, the lips of the os uteri appeared somewhat thicker and softer, and more pink in colour than usual; and a round cyst about the size of a small pea, and of a leaden hue, was found in the womb closely and organically attached to it, just below the opening of the larger Fallopian tube, by a fibrous base, the diameter of which was fully half of that of the cyst itself. This cyst which was filled with fluid blood, was also composed of dense fibrous tissue, and became quite white like the texture of the uterus when the blood contained in it was washed away.

